# Unclassified hepatocellular adenoma with beta-catenin mutation: a case report

**DOI:** 10.1186/s40792-021-01131-9

**Published:** 2021-02-12

**Authors:** Ryo Muranushi, Kenichiro Araki, Norifumi Harimoto, Takehiko Yokobori, Kouki Hoshino, Kei Hagiwara, Norihiro Ishii, Mariko Tsukagoshi, Takamichi Igarashi, Akira Watanabe, Norio Kubo, Shinichi Aishima, Ken Shirabe

**Affiliations:** 1 Department of General Surgical Science, Division of Hepatobiliary and Pancreatic Surgery, Graduate School of Medicine, Gunma University, 22 Showa-Machi, Maebashi, Gunma 371-8511 Japan; 2 Division of Integrated Oncology Research, Gunma University Initiative for Advanced Research (GIAR), Maebashi, Japan; 3 Department of Pathology and Microbiology, Saga University, Saga, Japan

**Keywords:** Hepatocellular adenoma, β-Catenin mutation, Hepatectomy

## Abstract

**Background:**

Hepatocellular adenoma (HCA) subtypes are considered as risk factors for malignant transformation; thus, an accurate diagnosis is important. We report a case of resected HCA previously diagnosed as unclassified HCA using immunohistochemistry, subsequently discovered to harbor a mutation in exon 3 of the beta (β)-catenin gene using deoxyribonucleic acid (DNA) sequencing.

**Case presentation:**

The patient was a 26-year-old woman who was referred to our hospital because of a 150-mm tumor in the right lobe of the liver. Considering the possibility of malignancy, we performed right lobe hepatectomy. Based on the histopathological and immunohistochemical findings, the tumor was diagnosed as an unclassified HCA. Next, we performed sequencing of DNA isolated from the tumor and identified a mutation in exon 3 of β-catenin, suggesting that the tumor contained an activating mutation of the β-catenin gene.

**Conclusion:**

β-Catenin mutations in HCA cannot be detected by immunohistochemistry alone, and molecular analysis is required to accurately diagnose and evaluate its prognosis.

## Background

Hepatocellular adenoma (HCA) is a rare, benign tumor of the liver, often observed in young women taking oral contraceptives, with an estimated incidence of 3 per 1,000,000 per year [1]. HCA is divided into four subtypes based on molecular and pathological features: hepatocyte nuclear factor 1 alpha (HNF1α)-mutated HCA (HHCA), inflammatory HCA (IHCA), β-catenin-mutated HCA (b-HCA), and unclassified HCA (UHCA) [2]. Immunohistochemical analysis usually shows a lack of liver fatty acid-binding protein (LFABP) in HHCA, serum amyloid A (SAA) and C-reactive protein (CRP) overexpression in IHCA, diffuse and strong glutamine synthetase (GS) expression and nuclear β-catenin in b-HCA, and no specific features in UHCA [3]. Because b-HCA is associated with a high risk of malignant transformation, accurate diagnosis of the subtype is important for evaluating its prognosis [4]. However, β-catenin mutation cannot be detected by immunohistochemical analysis alone [5].

Herein, we report a case of UHCA that was demonstrated to have β-catenin mutation and discuss the significance of the collaboration between immunohistochemistry and molecular analysis in the diagnosis of HCA.

## Case presentation

**Fig. 1:**
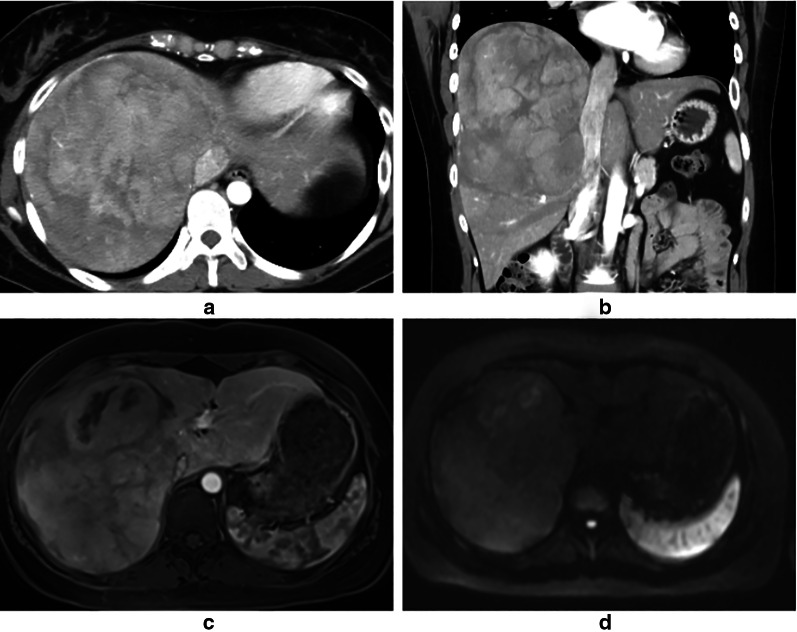
Enhanced computed tomography (CT) and gadolinium ethoxybenzyl diethylenetriamine pentaacetic acid-enhanced MRI findings CT showed a 150-mm-diameter tumor in the right hepatic lobe: **a** axial view, **b** coronal view. MRI showed **c**. No high intensity is detected compared to the background liver in the diffusion-weighted images (**d**)

**Fig. 2:**
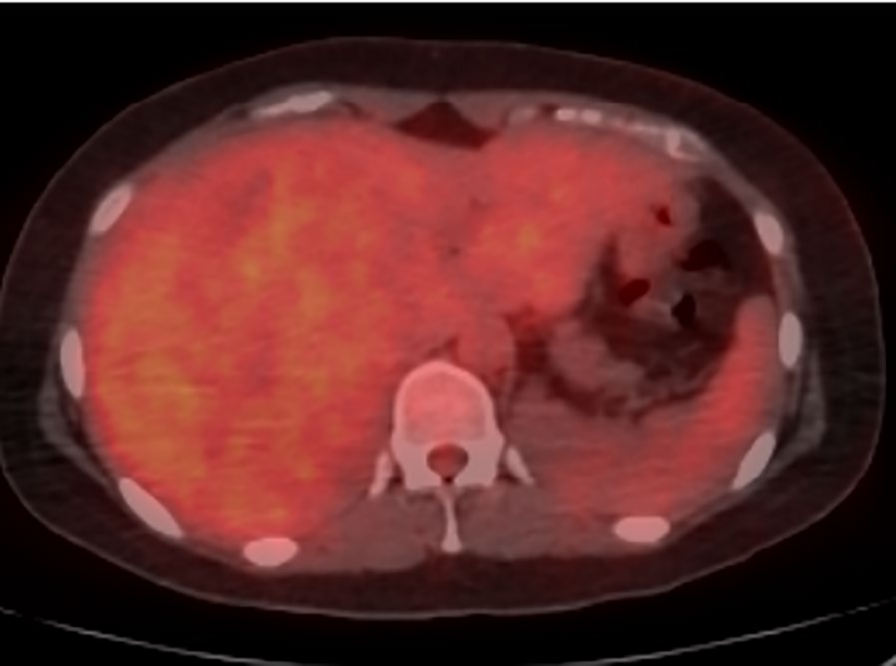
18F-fluorodeoxyglucose positron-emission tomography (FDG-PET) finding. FDG-PET showed slightly high FDG uptake of the tumor (SUVmax = 2.7). *FDG* 2-fluoro-2-deoxy-d-glucose, *SUVmax* maximum standard unit value

**Fig. 3:**
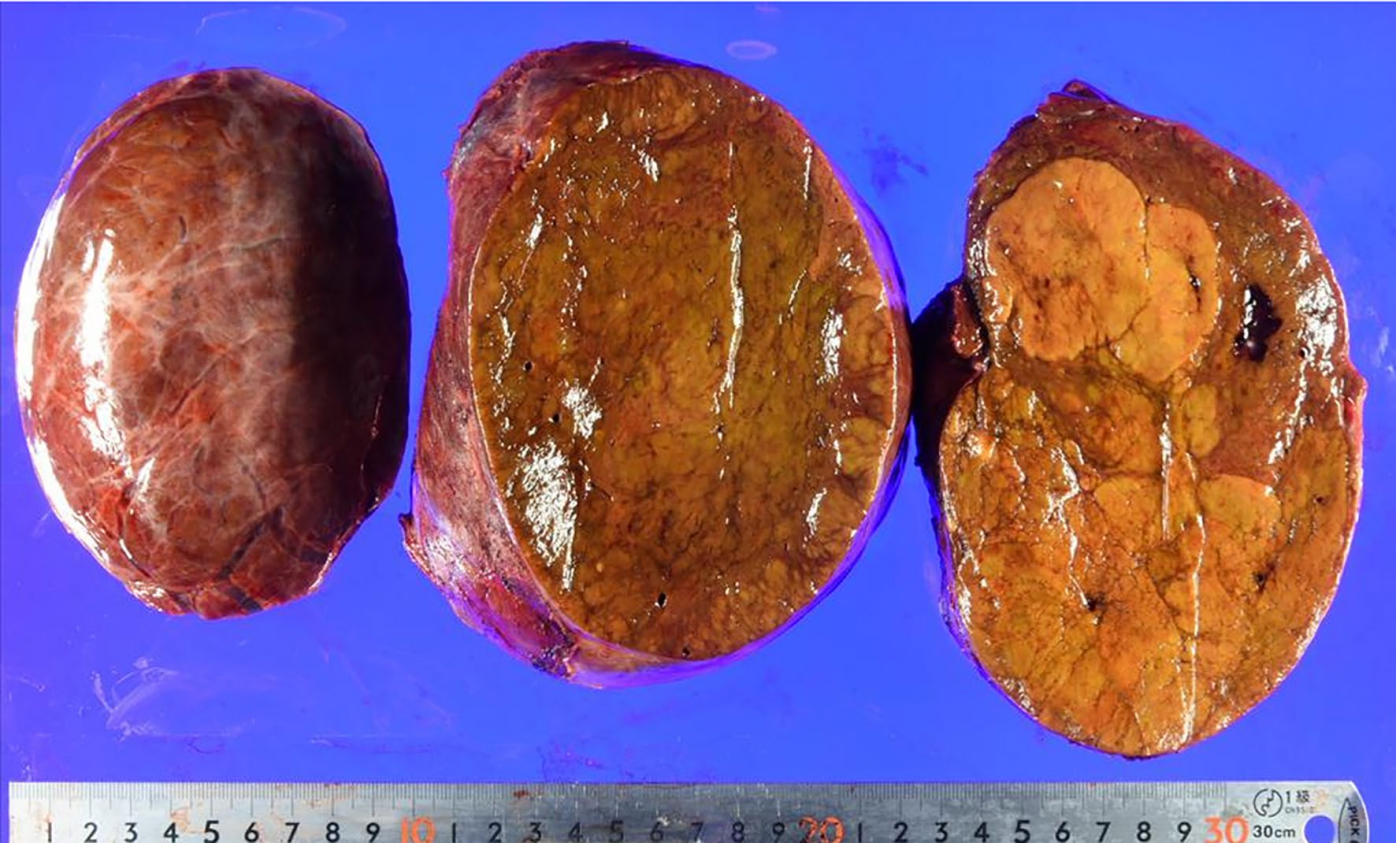
Photograph of the resected specimen. The tumor measuring 155 × 140 mm in the resected right lobe

**Fig. 4:**
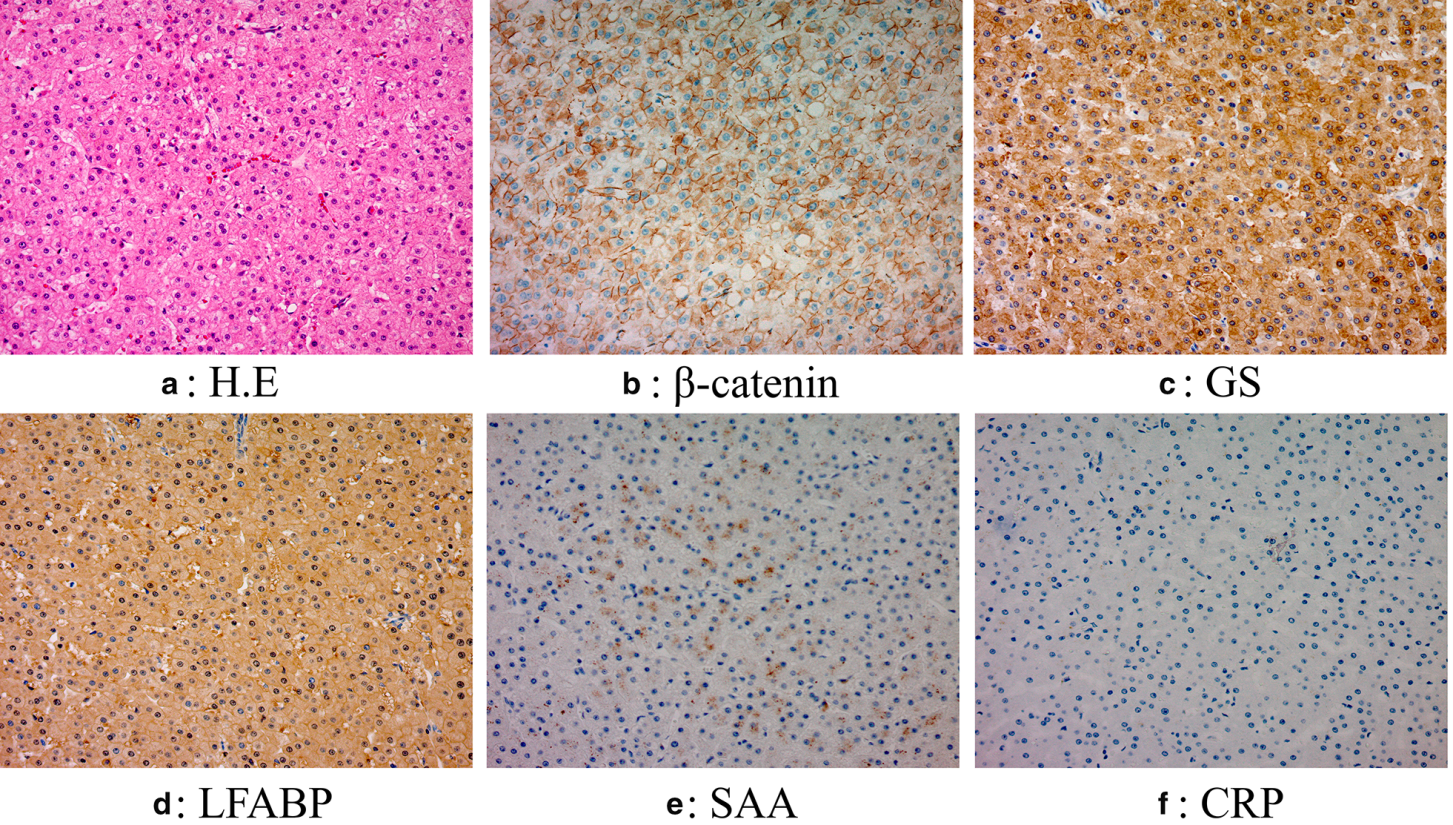
Histopathology and immunohistochemistry findings. Hepatocellular cells with poor atypia proliferated diffusely in the tumor (**a**) H&E staining (magnification, 200 ×). Immunohistochemical analysis shows positive β-catenin in the cellular membrane and negative in the nucleus (magnification, 200 ×) (**b**), diffuse GS (magnification, 200 ×) (**c**), LFABP is positive (magnification, 200 ×) (**d**), SAA is negative (magnification, 200 ×) (**e**), and CRP is negative (magnification, 200 ×) (**f**). H&E stain, hematoxylin and eosin staining; GS, glutamine synthase; LFABP, liver fatty acid-binding protein; SAA, serum amyloid A; CRP, C-reactive protein

**Fig. 5:**
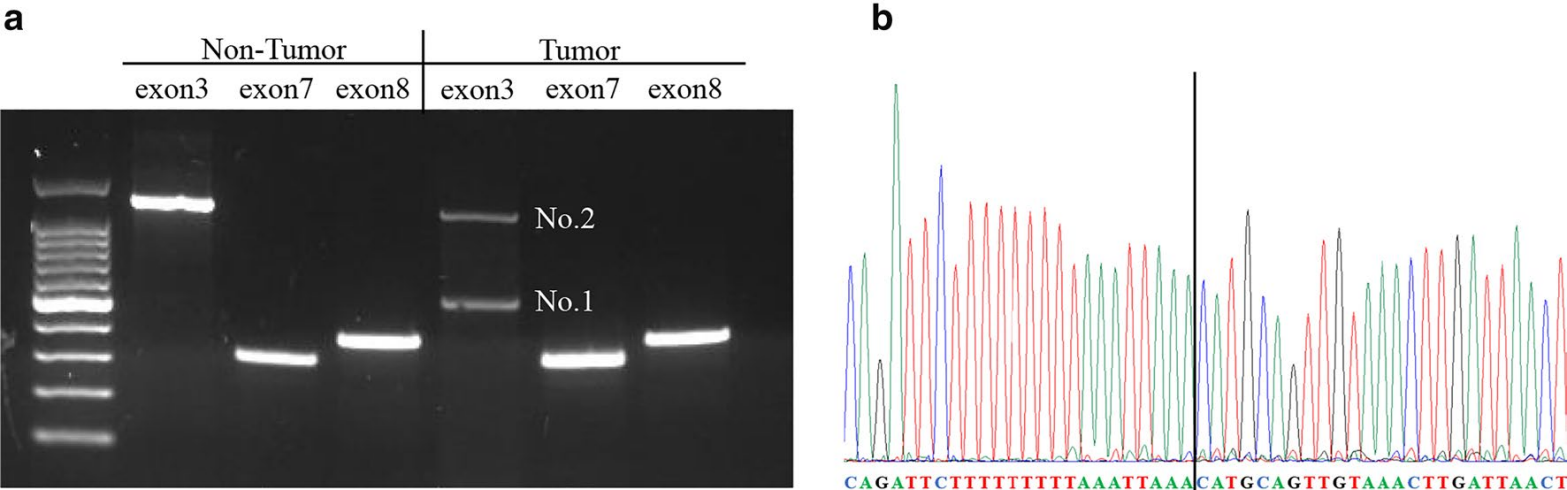
PCR and direct DNA sequencing results. **a** Electrophoresis of PCR products for exon 3, exon 7, and exon 8 of the β-catenin gene in tumor and non-tumor tissues. Leftmost is a 100-bp DNA ladder (Takara Bio Inc., Shiga, Japan). The PCR products of exon 3 of the β-catenin gene amplified from tumor DNA show two different bands, while that from non-tumor DNA shows a single band. The same band length is detected in exon 7 and exon 8. **b** DNA sequencing of exons 3 shows a large deletion, including introns between exon 2 and exon 3 to exon 4. The deletion site is marked by the solid black vertical line. PCR, polymerase chain reaction; DNA, deoxyribonucleic acid

The patient was a 26-year-old woman. She was admitted to our hospital because of a massive hepatic tumor, which was detected incidentally during investigations for hepatic disorders. She had no history of oral contraceptives. Hepatitis B virus surface antigen and hepatitis C virus antibody were both found to be negative. The patient’s blood tests showed aspartate aminotransferase, 26 U/L (normal range, 5–30 U/L); alanine phosphatase, 14 U/L (normal range, 10–30 U/L); gamma-glutamyl transpeptidase, 260 IU/L (normal range, 10–47 IU/L); total bilirubin, 0.7 mg/dL (normal range, 0.2.1.2 mg/dL); carcinoembryonic antigen, 0.9 ng/mL (normal range, < 5.0 ng/mL); carbohydrate antigen 19–9, 9.0 U/mL (normal range, < 15 U/mL); alpha-fetoprotein, 1.1 ng/mL (normal range, < 15 ng/mL); and protein induced by vitamin K absence or antagonist II, 192 mAU/mL (normal range, < 15 mAU/mL). An indocyanine green retention rate at 15 min was 4.2% with Child–Pugh grade A. Enhanced computed tomography showed a 150-mm-diameter enhanced tumor on the right hepatic lobe (Fig. 1a, b), with a clear border between the tumor and the peripheral tissue. Gadolinium-enhanced magnetic resonance imaging also showed an enhanced tumor (Fig. 1c), with no intensity difference compared to the background liver in the diffusion-weighted images (Fig. 1d). ^18^F-fluorodeoxyglucose positron-emission tomography showed a slightly high 2-fluoro-2-deoxy-d-glucose uptake in the tumor (maximum standard unit value = 2.7) (Fig. 2). Based on these findings, we performed right lobe hepatectomy because malignancy, including hepatocellular carcinoma (HCC), could not be excluded. The tumor was yellowish-brown, with demarcating borders, measuring 155 × 140 mm (Fig. 3). Histopathological findings showed diffuse proliferation of the hepatocellular cells, with poor atypia (Fig. 4a). Although some tumor cells had doublet nuclei, no difference in nuclear size, nuclear chromatin, or karyomitosis was observed. Based on these findings, a diagnosis of hepatocellular adenoma was made. Immunohistochemical analysis was positive for membranous β-catenin, negative for nuclear β-catenin, diffusely positive for GS, positive for LFABP, and negative for SAA and CRP (Fig. 4b, c, d, e, f, respectively). The non-tumor tissue showed similar membranous β-catenin staining (Additional file 1: Figure S1). Based on these findings, the tumor was diagnosed as UHCA. Next, we extracted DNA from the tumor and non-tumor tissues using the DNeasy Blood and Tissue Kit (QIAGEN, Germany) and performed DNA polymerase chain reaction (PCR) using primers to amplify β-catenin (CTNNB1) exons 3, 7, and 8 (Table 1). The primers designs were referenced from Rebouissou et al. [6]. The primers of exon 3 were designed to detect sequences between exon 2 and exon 4. Electrophoresis of PCR products from exon 3 showed two different bands (No. 1 and No. 2) from the tumor and one band from non-tumor DNA (Fig. 5a). The same length bands were detected in exon 7 and 8 (Fig. 5a). We extracted the DNA from each gel, including the bands, and performed direct DNA sequencing. The amplicon of No. 1 band (approximately 530-bp) was detected only from the tumor tissue but not from the non-tumor tissue and showed a large deletion of 631 bp from the intron between exon 2 and exon 3 to exon 4 (Fig. 5b; The borderline indicates the deletion site.). Based on these results, we concluded that the tumor had a β-catenin mutation. The amplicon with a band around 1100 bp (No. 2) was considered to be contaminated from non-tumor tissue. There was no mutation in exon 7 and 8 of the tumor. According to these findings, the tumor was demonstrated to have a mutation that had a large deletion including the beta-transducin repeats-containing protein (β‐TrCP) binding site.

**Table 1:** The primers list for exon 3, exon 7, and exon 8 of the catenin beta-1 gene

Exon	Primer sequence (5′ to 3′)
Exon 3 forward	GGTATTTGAAGTATACCATAC
Exon 3 reverse	CTGGTCCTCGTCATTTAGCAG
Exon 7 forward	GGTTGGTAATATGGCTCTTCT
Exon 7 reverse	CAGTAGTTAAAGTTCTACCACC
Exon 8 forward	TGGCAAAGTGAAGGAAACTG
Exon 8 reverse	CAAGGAGACCTTCCATCCC

The postoperative course was good, and the patient was discharged 13 days after the surgery. No recurrence has been observed for 1 year.

## Discussion

UHCA accounts for 10% of all HCAs and has not been characterized by a specific phenotype, radiological features, or genetic mutations [7]. The significance of β-catenin mutation in UHCA has not been established.

B-HCA accounts for 7–14% of HCA and is characterized by activating mutations of the CTNNB1 gene [7]. Activating mutations can occur in exons 3, 7, or 8 of the CTNNB1 gene in HCC and HCA [8] and are found in 20–34% of HCCs, suggesting that β-catenin is the most frequently activated oncogene in HCC [9].

Several mutations have been identified in the CTNNB1 gene in HCA, leading to the activation of β‐catenin. Large in‐frame exon 3 deletions and point mutations occurring at the D32‐S37 residues cause a strong β‐catenin signal due to the direct disruption of the β‐TrCP binding site, consequently suppressing its ubiquitination and proteasomal degradation [8]. Male sex and β-catenin exon 3 mutations in b-HCA are significantly associated with a higher risk of malignant transformation [10]. Mutations located at the T41 amino acid residue show moderate activation, whereas mutations located at S45, K335, and N387 show weak activation of β‐catenin [6]. Mutations in exon 3 often show both homogeneous GS overexpression and nuclear β‐catenin accumulation [6]. Conversely, exon 3 S45, exon 7, and exon 8 mutations demonstrate only weak, heterogeneous GS expression without nuclear β‐catenin accumulation [11]. β-Catenin mutations are not associated with nuclear β-catenin staining, suggesting that some CTNNB1 alterations may not disrupt GS and β-catenin patterns.

In our patient, although the tumor had a large deletion of exon 3, including the β‐TrCP binding site, it did not show nuclear β-catenin staining, leading to UHCA diagnosis. Because both tumor and non-tumor sections showed similar membranous β-catenin staining, it was not suggested that the large deletion of CTNNB1 gene caused the loss of β-catenin antigenicity.

Regarding cases with discordance between GS and β-catenin staining, the tumors are speculated to have activation of an alternative pathway causing GS expression [4]. Austinat et al. reported a case of HCC that showed an expression of cytosolic β-catenin and GS, which was identified as a 3-bp deletion affecting the tyrosine at position 97 within the adenomatous polyposis coli protein-binding site of Axin1 [12]. Additionally, another study described strong GS staining in a peliotic HCA, suggesting that vascular flow alterations and hepatic parenchymal remodeling increased GS expression [13]. Therefore, aberrant GS expression can occur without β-catenin nuclear translocation.

Abnormal GS expression is considered to be a useful marker for b-HCA, even in the absence of aberrant nuclear β-catenin staining [14]. In HCC, GS immunostaining was reported to have better sensitivity and specificity than β‐catenin because β‐catenin antibody is often difficult to interpret due to weak staining and lower sensitivity [15, 16]. In cases with positive GS staining, molecular analysis is needed to detect β-catenin mutations. In this case because the tumor possibly includes a strong activation of β-catenin signaling, close follow-up is necessary based on the risk of recurrence.

## Conclusion

We performed hepatectomy for HCA, and β-catenin mutation was detected using PCR and direct DNA sequencing. It is necessary to perform mutational analysis in cases with positive GS staining.

## Supplementary Information

**Additional file 1: Figure S1.** Immunohistochemistry findings of β-catenin in non-tumor tissue. β-Catenin is positive in the cellular membrane and negative in the nucleus.

## Data Availability

Data sharing is not applicable to this article as no datasets were generated or analyzed during the current study.

## Abbreviations

HCA: Hepatocellular adenoma
HHCA: Hepatocyte nuclear factor 1 alpha-mutated HCA
IHCA: Inflammatory HCA
b-HCA: β-Catenin-mutated hepatocellular adenoma
UHCA: Unclassified hepatocellular adenoma
LFABP: Liver fatty acid-binding protein
SAA: Serum amyloid A
CRP: C-reactive protein
GS: Glutamine synthetase
HCC: Hepatocellular carcinoma
DNA: Deoxyribonucleic acid
CTNNB1: β-Catenin
PCR: Polymerase chain reaction
β‐TrCP: Beta-transducin repeats-containing proteins
H&E staining: Hematoxylin and eosin staining
MRI: Magnetic resonance imaging
