# Supplementary figures and images for: Unclassified hepatocellular adenoma with beta-catenin mutation: a case report
Source: Surg Case Rep. 2021 Feb 12;7:46. doi: 10.1186/s40792-021-01131-9 (PMC7881073; doi:10.1186/s40792-021-01131-9)

## Slide 1
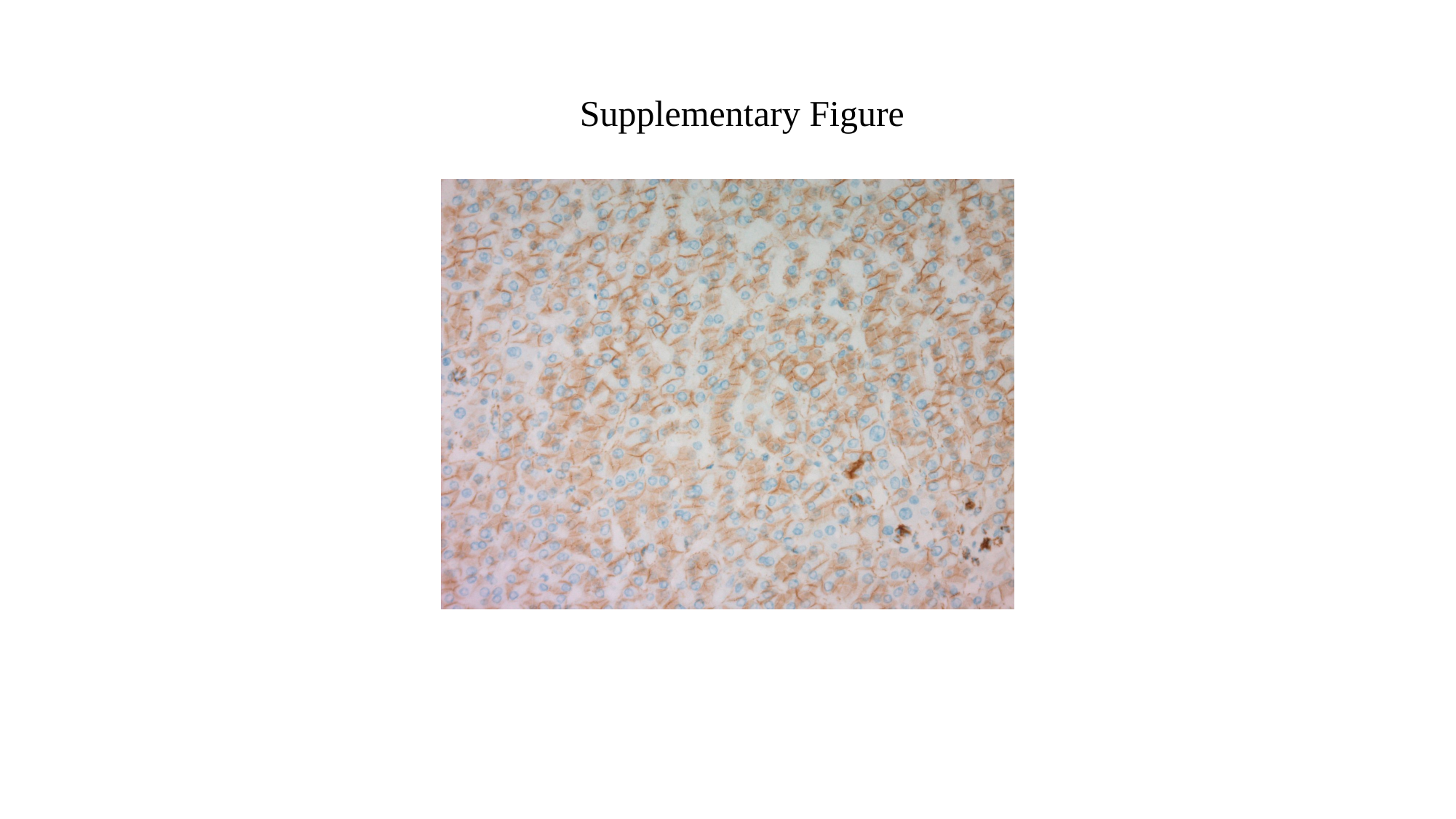

Supplementary Figure

Supplement: Supplementary file 1 — Additional file 1: Figure S1. Immunohistochemistry findings of β-catenin in non-tumor tissue. β-Catenin is positive in the cellular membrane and negative in the nucleus. [file 40792_2021_1131_MOESM1_ESM.pptx]
